# Long-term surveillance of the feline leukemia virus in the endangered Iberian lynx (*Lynx pardinus*) in Andalusia, Spain (2008–2021)

**DOI:** 10.1038/s41598-024-55847-3

**Published:** 2024-03-05

**Authors:** Fernando Nájera, Guillermo López, Tere del Rey-Wamba, Rimsha A. Malik, Germán Garrote, Marcos López-Parra, Leonardo Fernández-Pena, Maribel García-Tardío, Rafael Arenas-Rojas, Miguel A. Simón, Irene Zorrilla, Isabel Fernández, Eva M. Alcaide, Carmen Ruiz, Luis Revuelta, Javier Salcedo, Regina Hofmann-Lehmann, Marina L. Meli

**Affiliations:** 1 Wildlife Health Center, School of Veterinary Medicine, University of California, Davis, CA 95616 USA; 2 Department of Animal Physiology, Faculty of Veterinary Medicine, Complutense University of Madrid, 28040 Madrid, Spain; 3 Asistencia Técnica de la Dirección General del Medio Natural y Desarrollo Sostenible de la Junta de Comunidades de Castilla-La Mancha, Plaza del Cardenal Siliceo s/n, 45071 Toledo, Spain; 4 Agencia de Medio Ambiente y Agua de Andalucía, C/ Johan G. Gutenberg 1, Isla de la Cartuja, 41092 Seville, Spain; 5 Department of Ecology and Evolutionary Biology, Princeton University, Princeton, NJ 08544 USA; 6 Consejería de Sostenibilidad, Medio Ambiente y Economía Azul, avda. Manuel Siurot, nº 50, 41013 Seville, Spain; 7 Clinical Laboratory, Department of Clinical Diagnostics and Services, and Center for Clinical Studies, Vetsuisse Faculty, University of Zurich, 8057 Zurich, Switzerland; 8 Present Address: College of Veterinary Medicine, Cornell University, Ithaca, NY 14853 USA

**Keywords:** Ecology, Microbiology, Zoology, Diseases

## Abstract

Feline leukemia virus (FeLV) infection is considered one of the most serious disease threats for the endangered Iberian lynx (*Lynx pardinus*) Over 14 years (2008–2021), we investigated FeLV infection using point-of-care antigen test and quantitative real-time TaqMan qPCR for provirus detection in blood and tissues in lynxes from Andalusia (Southern Spain). A total of 776 samples from 586 individuals were included in this study. The overall prevalence for FeLV antigen in blood/serum samples was 1.4% (5/360) (95% CI: 0.2–2.6), FeLV proviral DNA prevalence in blood samples was 6.2% (31/503) (95% CI: 4.1–8.6), and FeLV proviral DNA in tissues samples was 10.2% (34/333) (95% CI: 7–13.5). From a subset of 129 longitudinally sampled individuals, 9.3% (12/129) PCR-converted during the study period. Our results suggest that FeLV infection in the Andalusian population is enzootic, with circulation of the virus at low levels in almost all the sampling years. Moreover, since only one viremic individual succumbed to the infection, this study suggests that lynxes may therefore control the infection decreasing the possibility of developing a more aggressive outcome. Although our results indicate that the FeLV infection in the Iberian lynx from Andalusia tends to stay within the regressive stage, continuous FeLV surveillance is paramount to predict potential outbreaks and ensure the survival of this population.

## Introduction

Feline leukemia virus (FeLV) belongs to the family *Retroviridae*, subfamily *Orthoretrovirinae*, and genus *Gammaretrovirus*. It has a single-stranded RNA genome that becomes associated with the target cell through the fusion of the viral envelope with the cell membrane. It then releases the nucleocapsid with viral RNA into the cytoplasm, which is transcribed by the reverse transcriptase (RT) enzyme to DNA and then transported to the cell nucleus, where it is subsequently integrated into the cell genome to become a “provirus”^1^. During cell mitosis, the daughter cells inherit the provirus, indicating that the cat may remain infected for life in such cases^2,3^. FeLV is classified into subgroups A (the parent virus), B, C, D, and T based on their interference and in vitro host range properties. Subgroups B and D arise from the recombination of the FeLV-A envelope (env) and the env genes of endogenous FeLV or endogenous retroviruses in the genomes of domestic cats (ERV-DCs)^4,5^. Subgroups C and T possibly arise from mutations in the FeLV-A env gene^6^. The recombination or mutation of the env gene often alters the interference and host ranges of FeLVs by affecting their receptor usage^7^.

In the domestic cat, FeLV infection may be abortive, regressive, progressive, or focal^8,9^. After infection, the virus replicates in local lymphoid tissues. Only in abortively infected felids does no detectable virus replication take place. In most other individuals, a primary viremia occurs via monocytes and lymphocytes and the virus spreads all over the body^10^. If bone marrow cells become infected, a secondary viremia can occur, with FeLV-containing leukocytes and platelets appearing in the blood, resulting in the virus being detectable by immunofluorescent antibody (IFA) assay. Regressive infection occurs when a viremic cat is capable of clearing viremia. This usually happens in 2–16 weeks, and the information for virus replication (proviral DNA) remains present in bone marrow stem cells^10^. Otherwise, if viremia cannot be controlled in this period, a progressive infection occurs, and viremia persists for months or years until death^10,11^. Focal infection is characterized by a persistent atypical local viral replication (e.g., in mammary glands, bladder, eyes) that can lead to intermittent or low-grade production of antigen, and therefore, these cats can have weakly positive or discordant results in antigen tests, or positive and negative results may alternate, with negative proviral results^12^. Progressively infected cats shed infectious virus in body fluids, including saliva, nasal secretions, milk, urine, and feces^10^. Regressively infected cats are considered FeLV carriers. They do not shed FeLV, but upon reactivation, reoccurring virus shedding is possible, and they can act as an infection source. As FeLV provirus is integrated into the cat’s genome, it is unlikely to be fully cleared over time^11^.

One of the most relevant clinical challenges while working with this retrovirus refers to diagnostics. This challenge derives from the different outcomes of infection, which can vary over time depending on the balance between the virus and the host’s immune system^9^. The most frequently used detection methods for FeLV include the detection of free FeLV p27 antigen, viral RNA, and proviral DNA. Recently, a point-of-care anti-FeLV antibody assay has been marketed in Europe, although more data is needed to test its reliability in detecting abortive FeLV infections under field conditions^9,13^. A practical approach to FeLV diagnosis has been developed by Hofmann-Lehmann and Hartmann and it also includes an algorithm for the diagnosis of FeLV infection in a single cat, developed by the European Advisory Board on Cat Diseases (ABCD)^9^. Following Hofmann-Lehmann and Hartmann (2020) guidelines, a confirmed positive result for free FeLV p27 antigen would suggest that the cat is antigenemic and an FeLV shedder at the point of testing. Regarding the outcome of infection, this indicates that the individual is progressively infected with persistent antigenemia/viremia, or has a regressive infection with transient antigenemia/viremia. Detection of FeLV RNA by RT-qPCR in saliva can also be taken as a marker for antigenemia and the outcome of infection can be similarly interpreted. As for FeLV provirus qPCR, a positive result suggests that the cat has been exposed to FeLV and has developed either progressive or regressive infection; an additional test (e.g., blood proviral loads or p27 antigen determination) should be pursued to differentiate between these outcomes. The only method to diagnose a FeLV abortive infection is by testing for FeLV antibodies, although the clinical and epidemiological relevance is very low for this outcome of infection^9^.

FeLV exposure has been demonstrated in several wild felids species including Iberian lynx^14^ (*Lynx pardinus*), European wildcat^15^ (*Felis silvestris*), jaguar^16^ (*Panthera onca*), puma^17^ (*Puma concolor*) and the endangered puma subspecies, the Florida panther^17^ (*Puma concolor coryi*), bobcat^18^ (*Lynx rufus*), ocelot^19^ (*Leopardus pardalis*), oncilla^19,20^ (*Leopardus tigrinus*), jaguarundi^20^ (syn. *Puma yagouaroundi*; *Herpailurus yagouaroundi*), guigna^21^ (*Leopardus guigna*), Far-eastern leopard^22^ (*Panthera pardus orientalis*), Far-eastern leopard cat^22^ (*Prionailurus bengalensis euptilurus*), leopard cat^23^ (*Prionailurus bengalensis*), clouded leopard^24^ (*Neofelis nebulosa*), cheetah^25^ *(Acinonyx jubatus),* and sand cat^26^ (*Felis margarita*).

The Iberian lynx is the most threatened felid species in the world, and it is currently cataloged as “endangered” by the International Union for Conservation of Nature^27^. By the early twenty-first century, just two isolated populations totaling less than 100 individuals remained in Andalusia, Southern Spain: Sierra Morena and Doñana areas^28^. After 20 years of comprehensive European Union-funded conservation programs (including both in situ and ex situ measures), the longevity of the species now relies on the approximately 1600 individuals distributed across fifteen population nuclei in Spain and Portugal^29,30^. One of the most recognized threats to the species is the impact of infectious diseases, especially FeLV^31^. A FeLV outbreak was reported in the Coto del Rey nucleus of the Doñana Iberian lynx population in early 2007^32,33^. The nucleus was then composed of 14 individuals: five breeding pairs (5F:5M) and four subadults (2F:2M)^32^. The virus quickly spread throughout the entire nucleus during the mating season and, by March, one breeding female and all seven males were found to be viremic. Six months later, five of the males had died^32^. All five breeding females gave birth, although two litters were lost in the first month of life^32^. One of the females and her two cubs were found to be viremic and were removed from the wild to a quarantine station^32^. The female turned the virus into latency 6 months after the extraction and was then re-introduced into the area^32^. The rest of the individuals remained viremic in captivity^32^. Since then, a specific FeLV Monitoring Campaign has been annually implemented in Andalusia, including both p27 ELISA and provirus qPCR testing in all lynxes found dead and all lynxes handled (including a sampling of the population for health monitoring)^32^. Due to the relevance of this infectious disease in Iberian lynx populations, the FeLV Monitoring Campaign is also carried out in new reintroduction areas of the Iberian Peninsula^34^.

The objectives of our study were to: (1) determine the prevalence of FeLV infection in Iberian lynx populations, (2) determine potential risk factors associated with FeLV infection in this species and (3) evaluate the dynamics of FeLV infection in longitudinally sampled animals during the study period, as well as from those individuals involved in the 2007 FeLV outbreak.

## Results

### FeLV prevalence and spatial clustering

During 2008–2021, the overall prevalence of (1) FeLV p27 antigen in blood, (2) FeLV provirus in blood and (3) FeLV provirus in tissues were 1.4% (± standard error SE 0.62, CI: 0.2–2.6), 6.2% (SE: ± 1.07, CI: 4.1–8.3), and 10.2% (± 1.66, 7.0–13.5), respectively (Table 1). Prevalence of antigenemia was higher in Doñana (2.4%; SE: ± 1.38; CI: 0–5.1) than in Sierra Morena (0.9%; SE: ± 0.61; CI: 0–2.1; χ^2^ = 1.4, *p* = 0.24) though not statistically significant while FeLV provirus positivity in blood was similar in both populations (6.5%; SE: ± 1.97; CI: 2.6–10.3 in Doñana and 6.1%; SE: ± 1.29; CI: 3.6–8.7 in Sierra Morena; χ^2^ = 0.02, *p* = 0.89). FeLV provirus prevalence in tissues was lower in Doñana (8.3%; SE: ± 3.02; CI: 2.4–14.2) than in Sierra Morena (11.1%; SE: ± 2.01; CI: 7.1–15; χ^2^ = 0.5, *p* = 0.48), though not statistically significant. The overall prevalence of FeLV provirus in blood in the 81 sympatric feral cats sampled in 2011–2019 was 14.8 (SE: ± 4.0%; CI: 7.1–22.6), and prevalence was significantly lower in Doñana (3.3%; SE: ± 3.3; CI: 0–9.8) than in Sierra Morena (21.6%; SE: ± 5.8; CI: 10.3–32.9); Z = 5.0, *p* = 0.026).

**Table 1:** Prevalence (in percentage of positive individuals), 95% confidence intervals, and sample size (between brackets) of FeLV p27 ELISA in blood, FeLV PCR proviral DNA in blood and tissues recorded in the free-ranging Iberian lynx population from Andalusia in 2008–2021.

Population	Prevalence (95% CI) [N]		
	ELISA p27 blood	PCR proviral DNA blood	PCR proviral DNA tissues
Doñana-Aljarafe	2.4 (0–5.1) [124]	6.5 (2.6–10.3) [155]	8.3 (2.4–14.2) [84]
Sierra Morena	0.9 (0–2.1) [231]	6.1 (3.6–8.7) [343]	11.1 (7.1–15) [244]
Dispersers	0 (0–0.43) [5]	0 (0–0.43) [5]	0 (0–0.43) [5]
Sex			
Male	2.1 (0.1–4.1) [190]	8.2 (4.8–11.6) [256]	14.4 (8.9–19.8) [160]
Female	0.6 (0–1.7) [170]	4.1 (1.6–6.6) [245]	6.2 (2.5–9.9) [162]
Age			
Juvenile	0 (0–0.04) [90]	1.7 (0–4.1) [117]	1.9 (0–5.5) [53]
Subadult	1.8 (0–4.2) [114]	4.9 (1.8–8) [185]	7.1 (3.1–11.1) [155]
Adult	2.1 (0–4.5) [140]	9.1 (4.9–13.4) [175]	16.8 (9.3–24.4) [95]
Older adult	0 (0–0.2) [15]	16.7 (1.8–31.6) [24]	22.7 (5.2–40.2) [22]
Overall	1.4 (0.2–2.6) [360]	6.2 (4.1–8.3) [503]	10.2 (7–13.5) [333]

**Figure 1:**
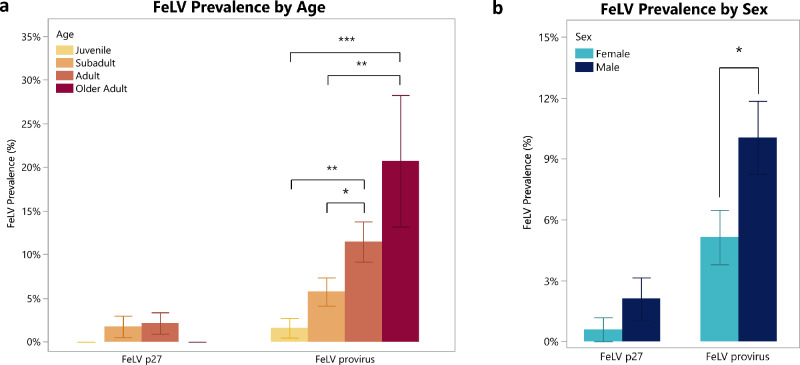
FeLV prevalence results by age (**a**) and sex (**b**). *p < 0.05, **p < 0.01, ***p < 0.001. Error bars denote ± SE.

Significant age and sex-related differences were found for the FeLV provirus positive tested lynxes: adult, older adult, and male lynxes were significantly more often positive (adult-subadult, χ^2^ = 4.21, *p* = 0.04; adult-juvenile, χ^2^ = 10.6, *p* = 0.001; older adult-subadult, χ^2^ = 8.1, *p* = 0.004; older adult-juvenile, χ^2^ = 17.6, *p* < 0.0001; male versus female, χ^2^ = 4.73, *p* = 0.03) (Fig. 1), whereas no statistical difference was present when antigenemia was evaluated (χ^2^ = 1.51, *p* = 0.22). The risk ratio (i.e., relative risk, RR) for FeLV infection in males was 1.96, while the RR for FeLV infection in adults was 2.2.

**Figure 2:**
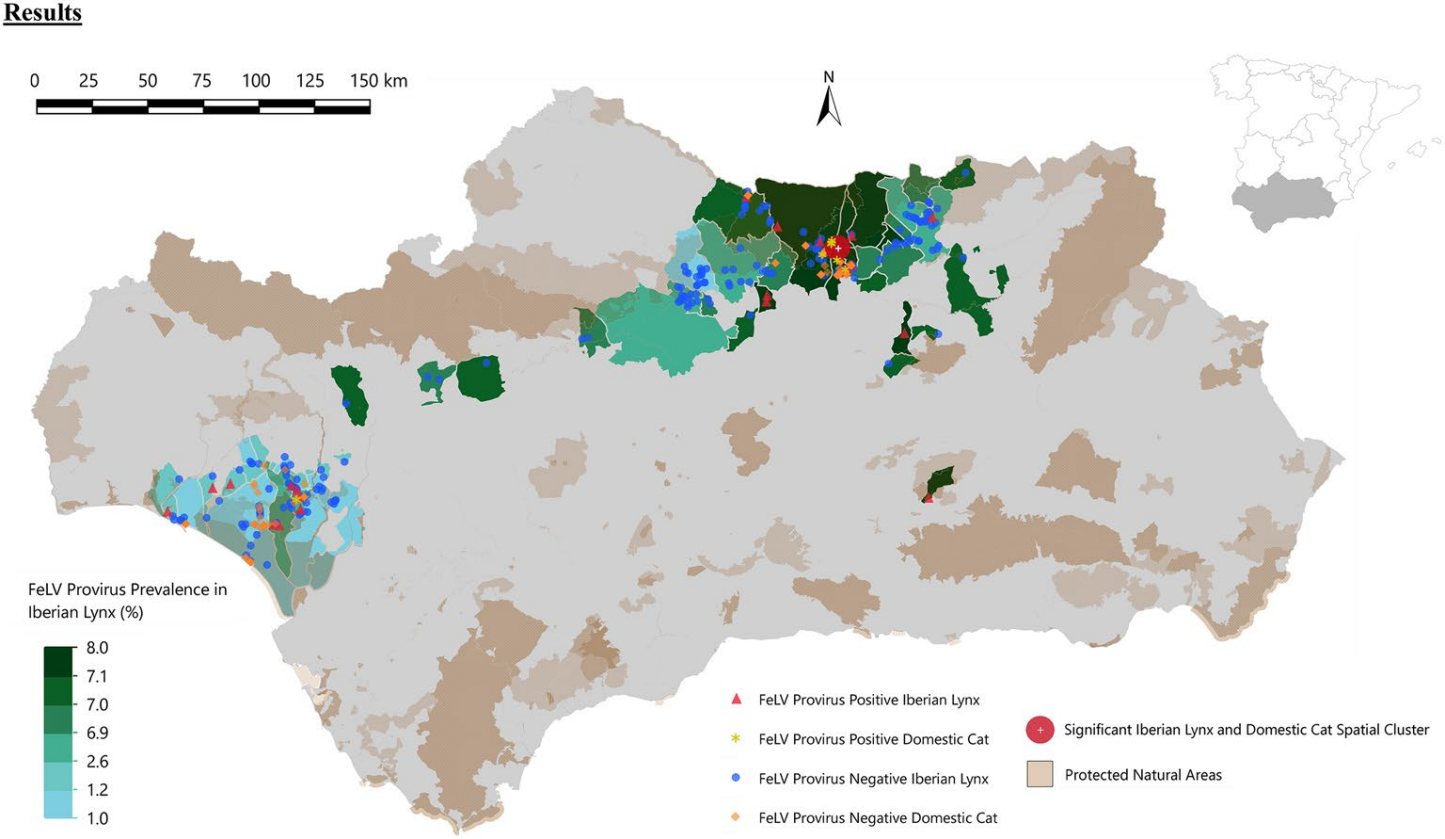
Map of Andalusia (southern Spain) displaying a FeLV provirus prevalence gradient reflecting 2008–2021 qPCR results at the municipal level in blue-green as well as nationally protected natural areas in brown. The Sierra Morena (northern range) population encompasses a significant spatial cluster for FeLV provirus-positive Iberian lynxes and domestic cats while the Doñana-Aljarafe population (western range) does not. Red triangles and blue circles indicate FeLV provirus positive and negative lynxes, respectively. Yellow stars and orange diamonds indicate FeLV provirus-positive and negative cats, respectively.

Results from our spatial data analysis revealed evidence of a significant spatial cluster of FeLV infection in the cats and lynxes from Sierra Morena (radius = 5.04 km; RR = 16.75; expected cases = 1.82; log-likelihood ratio = 14.65; *p* = 0.00023) (Fig. 2).

### FeLV prevalence by years

**Figure 3:**
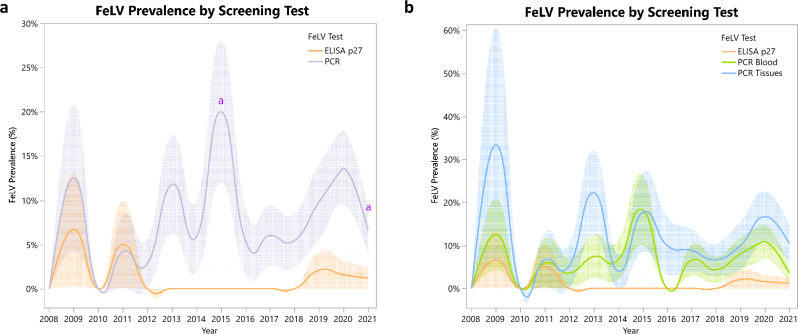
FeLV prevalence by year taking into account each of the screening methods used in this study. Letters indicated significant pairwise differences between years (*p* < 0.05). Shaded error bar denotes ± SE.

**Figure 4:**
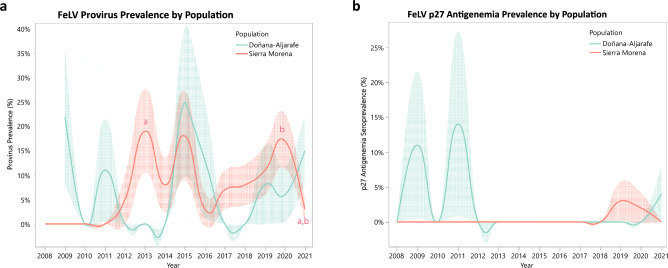
(**a**) FeLV prevalence results by population using FeLV proviral DNA in blood and tissue as screening method. Letters indicated significant pairwise differences between years (*p* < 0.05). (**b**) FeLV antigenemia prevalence results by population using ELISA p27 as screening method. Shaded error bars denote ± SE.

Results obtained during the FeLV surveillance by year (2008–2021) are depicted in Figs. 3 and 4.

The mean annual FeLV p27 prevalence ranged from 0–6.67%, while the mean FeLV provirus prevalence ranged from 0–18.8% in blood and 0–33.3% in tissue. Pairwise Fisher’s exact tests revealed very few significant maxima and minima in FeLV prevalence over time. Significant provirus differences were found for both populations combined (Fig. 3) between 2010–2015 (*p* = 0.019) for Sierra Morena (Fig. 4) between 2013- 2021 (*p* = 0.03) and 2020–2021 (*p* = 0.016).

### FeLV provirus qPCR performance and agreement in different tissues collected during necropsy

To explore the performance and agreement of the FeLV provirus qPCR in different organs and blood, we calculated the prevalence of positive PCR proviral DNA in a sub-sample (*n* = 33) of lynxes that were tested in more than one of the target tissue/blood collected upon necropsy and yielded a positive result for provirus qPCR in at least one compartment, 72.4% (SE: ± 8.3, CI: 56.1–88.7) tested positive in blood, 78.7% (SE: ± 7.1; CI: 64.8–92.7) resulted positive in bone marrow, 81.2% (SE: ± 9.8; CI: 62.1–100) were positive in intestine, 96.5% (SE: ± 3.4; CI: 89.9–100) resulted positive in mesenteric lymph node, and 47% (SE: ± 12.1; CI: 23.3–70.8) tested positive in spleen. Moderate to substantial agreement was found between PCR proviral DNA performed in blood and bone marrow [Kappa = 0.43 (SE: ± 0.19; CI: 0.06–0.81)], blood and intestine [Kappa = 0.81 (SE: ± 0.17; CI: 0.47–1)], bone marrow and intestine [Kappa = 0.81 (SE: ± 0.17; CI: 0.47–0.81)], and bone marrow and spleen [Kappa = 0.55 (SE: ± 0.22; CI: 0.11–0.99)].

### Longitudinal survey

Our longitudinal survey included 129 lynxes out of the 586 individuals sampled in this study (Table 2). The majority (90.6%; 117/129) of the lynxes remained negative at different sampling times. PCR conversion was observed in 9.3% (12/129) of the lynxes during the study period. Sampling intervals between negative and positive results ranged from 1 to 6 years, although we confirmed a conversion from PCR negative to positive within 4 weeks in one lynx. In one individual (Rayuela), the detection of FeLV proviral DNA performed in tissues (bone marrow and mesenteric lymph node) failed in its last sampling time, despite the detection of provirus DNA in blood for two consecutive years. This PCR reversion occurred within almost 4 years between samplings (Table 2).

**Table 2:** FeLV diagnosis by provirus qPCR in longitudinally sampled lynxes.

ID	Population^a^	Sex^b^	Sampling year (age at sampling)^c^													
			2008	2009	2010	2011	2012	2013	2014	2015	2016	2017	2018	2019	2020	2021
Bocacha	D	M	–	–	Neg (A)	–	–	–	–	Neg (A)	Pos (OA)	–	–	–	–	–
Cerrajero	SM	M	–	–	Neg (A)	–	–	Pos (OA)	–	–	–	–	–	–	–	–
Erodia	D	F	–	–	–	–	–	–	–	Neg (A)	–	–	–	–	–	Pos (OA)
Galilei*	SM	M	–	–	–	–	–	–	Neg-Pos (A)	–	–	–	–	–	–	–
Inci	SM	F	–	–	–	–	–	–	–	–	Neg (A)	–	–	–	–	Pos (A)
Kyler	D	M	–	–	–	–	–	Neg (J)	Neg (SA)	Pos (SA)	–	–	–	–	–	–
Libertad	SM	M	–	–	–	–	–	–	–	–	–	–	–	Neg (A)	Pos (A)	–
Querub	SM	M	–	–	–	–	–	–	–	–	–	–	–	Neg (J)	Pos (SA)	–
Quitu	SM	M	–	–	–	–	–	–	–	–	–	–	–	Neg (J)	Pos (SA)	–
Rapas	SM	F	–	–	–	–	–	–	–	–	Neg (OA)	Pos (OA)	–	–	–	–
Rayuela	D	F	Pos (A)	Pos (A)	–	–	–	Neg (A)	–	–	–	–	–	–	–	–
Viana	D	F	–	Neg (A)	–	–	Neg (A)	–	–	Pos (OA)	–	–	–	–	–	–

^a^*D* Doñana, *SM* Sierra Morena; ^b^*M* male, *F* female. (*J*) juvenile, (*SA*) subadult, (*A*) adult, (*OA*) older adult. – not tested; *PCR positive converted within the same month and year.

### Prevalence in sympatric domestic cats

Samples from 81 sympatric domestic cats from Sierra Morena (n = 51) and Doñana (n = 30) were screened for FeLV provirus in blood. The proportion of domestic cats that were FeLV positive in Sierra Morena was 21.6% (SE: ± 5.76; CI: 10.3–32.9) whereas in Doñana the proportion was significantly lower at 3.3% (SE: ± 3.28; CI: 0–9.8) (χ^2^ = 4.97, df = 1, *p* = 0.02). Overall, the FeLV provirus prevalence in domestic cats was 14.8% (SE: ± 8.95; CI: 7.1–22.6).

### Clinical and laboratory findings in antigenemic lynxes

Five (four males and one female) out of the 776 (0.6%) samples from 586 lynxes were antigenemic (ELISA p27 positive) during the study period: three in Doñana and two in Sierra Morena. Four of them (two from Doñana and two from Sierra Morena) were road-killed, and FeLV antigenemia was considered a necropsy incidental finding. No signs of disease were found in any of these four necropsied lynxes. One lynx sampled during a routine exam in Doñana in November 2011 was FeLV p27-positive by the POC ELISA test. The most relevant findings were mild anemia, hypoalbuminemia, hyperglobulinemia and high plasma levels of urea nitrogen (BUN), Aspartate Aminotransferase (AST) and Lactate Dehydrogenase (LDH). Blood was PCR-positive for provirus. Further analysis showed that the animal was negative for Feline Immunodeficiency Virus (FIV) provirus, Canine Distemper Virus (CDV), Feline Herpesvirus-1 (FHV-1), Feline Calicivirus (FCV), Feline Coronavirus (FCoV), Feline Parvovirus (FPV), *Bartonella* spp., *Chlamydophila felis* and *Cytauxzoon* spp. The animal also tested negative for antibodies against CDV, FHV and FCoV by ELISA, and for antibodies against FPV and FCV by indirect immunofluorescence analysis (IFA). Given its viremic status, it was placed in a wildlife rescue center. Despite ad libitum feeding, it progressively lost body condition and, after 2 months, became very weak. By mid-January, the individual presented with vomiting, diarrhea, and anorexia. The animal was handled for a clinical evaluation and no anesthesia was needed. Dehydration, tachycardia, and a severe non-regenerative anemia (7% hematocrit) were found. Antibiotics, steroids, and supportive care were provided; however, the lynx did not survive. The necropsy showed signs of severe bone marrow aplasia, septicemia by *Streptococcus sp*. and necrosis in kidneys and spleen.

### Outcome of lynxes involved in the outbreak of 2007

Results from FeLV screening at different periods, months of viremia (when applicable) and cause of death from lynxes involved in the 2007 FeLV outbreak from a subpopulation of Doñana are presented in Table 3. The cause of death for FeLV provirus positive individuals varied from road-killed to squamous cell carcinoma, though most deaths were attributed to bone marrow aplasia.

**Table 3:** FeLV p27 ELISA results obtained from lynxes involved in the 2007 FeLV outbreak.

Name	Sex	Age	ELISA p27 December 2006	ELISA p27 Spring 2007	ELISA p27 at time of death	PCR Provirus blood at the time of death	PCR Provirus tissues at the time of death	Months of antigenemia	Cause of death
Viciosa*	F	A	Negative	Negative (Provirus DNA positive)	Negative	Negative	Negative	N.A	Roadkill
Wari*	F	A	Negative	Negative	Negative	Negative	Negative	N.A	Dog attack
Rayuela	F	A	Negative	Positive	NA	NA	NA	5–12	Unknown
Teo*	F	A	Negative	Negative	Negative	Negative	Negative	N.A	Roadkill
Viana	F	A	Negative	Negative	Negative	Positive	Negative	< 3	Roadkill
Centaurea	F	SA	Negative	NA	Negative	Negative	Negative		Tuberculosis
Calendula	F	SA	Negative	NA					Unknown
Coca	M	SA	Negative	Positive	Negative	Positive	Positive	60	Squamous cell carcinoma
Roman	M	A	Positive	Positive	Positive	Positive	Positive	6–12	Bone marrow aplasia
Nati II	M	A	Negative	Positive	Positive	Positive	Positive	3	Bone marrow aplasia
Arrayan	M	A	Negative	Positive	Positive	Positive	Positive	2	Bone marrow aplasia
Uda	M	A	Negative	Positive	Positive	Positive	Positive	2	Bone marrow aplasia
Cicuta	M	SA	Negative	Positive	Positive	Positive	Positive	6	Bone marrow aplasia

*F* female, *M* male, *A* adult, *SA* subadult, *NA* not available. *Individuals that tested negative but were involved in the outbreak by means of spatial overlap and potential contact with positive lynxes.

One of the subadult males extracted from the field during the outbreak (“Coca”) was the only lynx kept in captivity for a longer period due to his viremic status. This individual remained viremic for almost 60 months. He was subjected to routine physical examinations to evaluate his FeLV condition. Results from these routine medical checkups are displayed in Table 4.

**Table 4:** FeLV diagnosis results from a subadult male (“Coca”) extracted from the field during the 2007 FeLV outbreak.

Examination date	ELISA p27	qPCR (proviral DNA)	RT-qPCR (viral RNA)
22/06/2007	Positive	Positive	NA
23/07/2007	Positive	Positive	NA
24/09/2007	NA	Positive	NA
18/03/2008	NA	Positive	NA
21/05/2008	NA	Positive	Positive
24/07/2008	Positive	Positive	NA
28/01/2009	NA	Positive	NA
26/05/2009	Positive	NA	Positive
20/07/2009	NA	Positive	NA
21/04/2010	Positive	Positive	NA
27/10/2010	Positive	Positive	Positive
04/05/2011	Positive	NA	NA
29/05/2012	Negative	Negative	NA
17/12/2013	Negative	Positive	Positive
07/08/2014	NA	Positive	Positive
09/11/2015	Negative	Positive	Negative
29/06/2017	Negative	Positive	NA
11/03/2020	Negative	Positive	Positive

*NA* not available.

## Discussion

To date, FeLV infection remains a disease threat not only for the Andalusian Iberian lynx population but also for the newly established populations in the Iberian Peninsula where it has caused morbidity and/or mortality following the reintroduction program^34^.

Due to the Iberian lynx’s role as an apex predator, initial FeLV exposure and infection of naïve lynx populations may derive from direct contact with, and killing of, free-roaming domestic cats whose populations significantly contribute to the FeLV reservoir and are considered to be the primary source of infection for lynxes^32,35,36^. This is in agreement with studies on other wild felids, where FeLV infection is reported to originate from domestic cats^18,37,38^. Therefore, areas with more free-roaming domestic cats may have higher FeLV prevalences and thus greater risk for FeLV spillover to lynxes. FeLV proviral prevalence in domestic cats from Sierra Morena is similar to the prevalence in domestic cats from lynx reintroduction areas in Southwestern Spain (9–23%), Andalusia (29.5%) or from previous studies in the remnant lynx populations from 2004–2006 (23%)^36,39,40^.

FeLV proviral DNA tissue prevalence in lynxes from Sierra Morena was higher than in lynxes from Doñana, although not statistically different. Accordingly, FeLV proviral prevalence in sympatric domestic cats from Sierra Morena was significantly higher than in Doñana and this was in accordance with owned cats tested at the national level (2.6% (1.4–4.8)^41^. Other factors that may have favored FeLV transmission and dissemination are: (1) time of FeLV infection (i.e., mating season, with higher interactions between males—including intraspecific fights—and higher male–female contacts), and (2) high density population nuclei which are thought to increase intraspecific contact^32^. The results of our study suggest that male Iberian lynxes have an increased risk of FeLV infection compared to females, which is in agreement with domestic cats, as males’ aggressive behavior plays a greater role than previously reported^8^. This is in accordance with other wild felids such as the guigna (*Leopardus guigna*), with males displaying more aggressive and daring behavior, being the dispersing sex and having larger home ranges than females, thus increasing infection probabilities^21,42^. Furthermore, in the studied population, adult and older adult lynxes have a significantly higher risk of FeLV infection than subadults and juveniles, which agrees with recent studies in domestic cats where adult cats were more likely to be FeLV-infected than juveniles^8^. The risk ratio suggests that males and adult lynxes are twice as likely to be FeLV infected than females and subadult lynxes, respectively. The FeLV spatial cluster detected in this study suggested that the virus spread relatively unevenly in Sierra Morena and that the probability of infection varies throughout this area when considering FeLV infection in both lynxes and domestic cats. The relative risk of infection is significantly higher within this identified spatial window when considering FeLV infection in both species.

This study highlights the relevance of FeLV proviral PCR testing as a sensitive method to detect infection in the Iberian lynx. It has been demonstrated that the detection of provirus by real-time qPCR is a more sensitive diagnostic marker for identifying FeLV-exposed domestic cats compared to previously used methods^43^. In our study, qPCR was > 4 times more sensitive in detecting FeLV exposure than antigen testing. This also has been shown in a pet cat population in Switzerland, where 6% of individuals in the study population were FeLV p27 positive and an additional 10% were provirus-positive with the absence of detectable antigenemia^43^. However, due to the risk that viremic individuals pose for the rest of the population and the management measures that need to be carried out when a antigenemic lynx is identified, we recommend continuing to perform ELISA p27 POC tests in all lynxes in parallel with the PCR proviral DNA, which while being sensitive in determining FeLV exposure, is limited in determining FeLV disease stages if provirus loads are not quantified^44,45^. In dead lynxes, quantifying provirus loads in tissues may help define the FeLV outcome, as described with experimental infected domestic cats^45^.

Regarding PCR proviral DNA performed in lynx tissues, this study showed that the mesenteric lymph node and intestine can be used as preferred target samples to detect FeLV infection in dead individuals. This is in agreement with Helfer-Hungerbuehler et al., where provirus loads were highest in the intestinal tract and lymphoid tissues in experimentally infected domestic cats^45^.

Based on frequent detections of FeLV throughout the study period, FeLV infection tends to be enzootic within the Andalusian lynx population. Prior to this study, FeLV circulation had already been demonstrated in this region (sampling years: 2004–2006)^39^.

From the longitudinal survey performed in lynxes involved in the 2007 outbreak that occurred in Doñana, the proportion of progressive infections found (38.4%) was likely due to increased host susceptibility to pathogens^32^, since the FeLV sequences isolated from that outbreak revealed their relationship with naturally occurring FeLV-A infections in domestic cats^46,47^. At that time, the loss of genetic diversity due to inbreeding in this region resulted in the apparent lack of acquired immunity and immunocompetence of the Iberian lynxes against infectious agents, especially FeLV^39,47–50^.

Since the 2007 outbreak which resulted in a high mortality rate due to bone marrow aplasia, there has not been a similar outbreak in the whole Andalusian Iberian lynx population. From 2008 to 2021, in Doñana only 2.4% (3/125) of individuals were viremic and only one succumbed to FeLV-related disease. Management to increase the genetic diversity in this population, such as translocations from Sierra Morena to Doñana^21,51,52^ have likely played a role in reversing the FeLV outcome in this region. In Sierra Morena, 0.9% (2/231) of animals were viremic and none of them presented clinical signs associated with FeLV at the time of testing. From our 2008–2021 longitudinal survey comprising 129 individuals, no viremic individuals were found, and just 9.3% were considered to be regressively infected. One individual seemed to be able to clear the infection. As observed in experimentally infected domestic cats, only about 8% of infected cats still harbored latent infection in different tissues 3 years post-viremia^8^. Another study observed one domestic cat that reverted from progressive to regressive after more than a year^53^. Without considering FeLV strain and/or infectious dose, both of which we could not investigate, our results suggest that lynxes may therefore control the infection within the bone marrow and other lymphoid tissues, limiting viremia and decreasing the possibility of developing a more aggressive disease due to progressive FeLV infection.

Although the results reported here suggest that the FeLV outcome in the Iberian lynx from Andalusia tends to stay within the regressive stage, continuous FeLV surveillance is paramount firstly to identify potential spillover (via predation) of FeLV-B from domestic cats co-infected with this recombinant strain that could have unpredictable effects for the Iberian lynx population, due to its lack of enFeLV^38^, and secondly to investigate the outcome of FeLV infection with synergistic co-infections, that may lead to more severe consequences.

Moreover, the endangered status of this felid species underscores the need to maintain disease surveillance in the entire Iberian lynx population as another conservation tool for the survival of the species.

## Methods

Sampling was performed throughout the range of the Iberian lynx in Andalusia, Southern Spain (Doñana and Sierra Morena populations; see Fig. 2) from January 2008 and December 2021. We tested for FeLV infection using ELISA for FeLV p27 antigen in blood and PCR for FeLV DNA provirus in blood and tissues at capture or necropsy. Lynx populations were monitored by combining both routine clinical evaluations of living lynxes performed in the framework of conservation programs (e.g., radio-tracking, health monitoring, translocations) and necropsies of dead individuals found by the mortality signal of the collar, direct citizen sightings (e.g., road-kills) or following an investigation (e.g., poached lynxes). A complementary study on sympatric domestic cats also included captures and carcass sampling.

### Fieldwork

Most living lynxes were captured using cage traps baited with a domestic rabbit^54^, whereas a few cases of injured individuals were captured using a blowpipe. Starting in 2015, trap monitors (Minkpolice^®^, Alert House ApS., Copenhagen, Denmark) were used and traps were checked within 30 min after the alarm activation^54^. Traps were always inactivated when the temperature exceeded 30 °C. Captured lynx were transferred to a transport and compression cage and transported to the Iberian lynx clinics (within 30 min). Once in the field clinic, a routine standardized handling and physical evaluation protocol was applied to all lynxes, which included blood collection. Up to 20 mL of blood was obtained by cephalic venipuncture and collected in EDTA-coated tubes, lithium heparin-coated tubes and serum separator tubes (Aquisel^®^ Barcelona, Spain). Blood collected in serum separator tubes was allowed to clot and was then centrifuged at 50×*g* for 15 min. The serum was removed and stored at − 20 °C until analysis. Three drops of whole blood were used in situ to run a fast (10 min) point-of-care (POC) antigen FeLV ELISA test (IDEXX^®^ Snap test, IDEXX Laboratories, Inc., Maine, USA), which detects the p27 protein of the FeLV capsid in blood. It is a very high sensitivity test to detect viremia [100% (CI: 96.9–100)]^55,56^, although it cannot detect latent infections (Table 5). Lynxes found p27 antigen positive (i.e., viremic) were transported to a wildlife rescue center. The rest of the individuals were safely released at the capture location after a clinical examination. Blood samples were refrigerated and sent to the Wildlife Laboratory of the Andalusia Regional Government (Centro de Análisis y Diagnóstico; CAD) for further analyses. Whenever a dead lynx was found, it was immediately transported to the CAD, where a complete necropsy was performed^31^. During necropsy, blood and tissue samples (bone marrow, intestine, mesenteric lymph nodes and/or spleen) were collected to evaluate FeLV infection along with other pathogens that may cause morbidity and/or mortality in the Iberian lynx. A POC FeLV p27 ELISA test (same as mentioned above) was also performed in dead animals when non-coagulated blood could be collected. In some instances, due to limited blood quantities or target tissue preservation, not all tests could be performed on all individuals.

**Table 5:** Possible outcomes of feline leukemia virus infection detected in this study and associated test results.

FeLV infection outcome	ELISA p27 antigen (blood)	PCR provirus (blood)	PCR provirus (tissues)
Progressive infection (formerly “persistent viremia”)	+	+	+
Regressive infection (with or without a previous “transient viremia”)	−*	+	+
No infection	−	−	−

*Only positive during transient viremia or after reactivation.

The sampling of sympatric domestic cats was mainly focused on the most anthropized areas of the lynx distribution range. Free-roaming domestic cats were trapped using a single guillotine-door, electro-welded-mesh cage traps baited with dead chickens and sardines. Domestic cat carcasses were found along roads due to road kills. In both free-roaming and dead cats, 1 mL of blood was collected from the cephalic vein and/or from cardiac puncture and placed in EDTA tubes. Blood samples were sent to the CAD for further analysis.

### Samples

A total of 776 records (from 586 lynxes) for FeLV screening were obtained, including 422 clinical evaluations and 353 necropsies (255 in Sierra Morena, 93 in Doñana, five dispersing individuals). During the study period, we longitudinally sampled 129 lynxes, obtaining a total of 329 records. Samples from 284 females, 288 males, and 14 individuals of undetermined sex were included in this study. We categorized the animals by age: 134 samples corresponded to juveniles (0–11 months old); 215 to subadults (1–2 years old); 199 to adults (3–10 years old), and 29 to older adults (11–16 years old). We could not determine the age of nine individuals. Overall, we performed FeLV p27 ELISA tests in 360 individuals (231 from Sierra Morena, 124 from Doñana, and five corresponding to dispersing individuals), FeLV proviral DNA in blood in 503 individuals (343 from Sierra Morena, 155 from Doñana, and five corresponding to dispersing individuals), and FeLV proviral DNA in one or several target tissues from 333 necropsied individuals (244 from Sierra Morena, 84 from Doñana and five dispersers).

Additionally, during the study period, 81 sympatric domestic cats (51 in Sierra Morena and 30 in Doñana; 14 carcasses and 67 live) were sampled (Fig. 2).

### Laboratory methods

Total nucleic acids were extracted from 200 μL EDTA-blood samples using the MagNA Pure LC Total Nucleic Acid Isolation Kit (Roche Diagnostics, Rotkreuz, Switzerland), or from tissues collected upon necropsy using the DNeasy and/or RNeasy Tissue kits (Qiagen, Hombrechtikon, Switzerland) according to the manufacturer’s instructions. FeLV provirus was analyzed through real-time TaqMan qPCR as described in Meli et al.^33^.

In antigenemic or ELISA p27 positive individuals, hematology and biochemistry panels were processed using EDTA-anticoagulated blood and serum, respectively, and analyzed using the BC-5000 Vet Mindray and a15 Biosystems, respectively.

Serological and/or molecular testing for Feline Immunodeficiency Virus (FIV)^57^, Canine Distemper Virus (CDV)^58^, Feline Herpesvirus-1 (FHV-1)^59^, Feline Calicivirus (FCV)^60^, Feline Coronavirus (FCoV)^61^, and Feline Parvovirus (FPV)^61^, *Bartonella* spp.^62^, *Chlamydophila felis*^63^*,* and *Cytauxzoon* spp.^33^ were conducted using methods described in respective literature.

### Statistical analyses

For statistical analysis, animals that had at least one positive result were considered positive. Multiple samples from the same animal were not used to avoid pseudo-replication and ensure data independence, and only the most recent sample was considered^64^. FeLV antigen prevalence and FeLV proviral DNA prevalence were estimated by dividing the number of positive animals by the total number of animals tested, using two-sided exact binomial 95% confidence intervals (CI).

Prevalence differences between sex, age, and population were tested using Pearson’s Chi-squared test. To analyze prevalence across years for populations and FeLV test type, we ensured the independence of time as autocorrelation of the times series was ruled out using Ljung–Box tests^65^. Subsequently, pairwise Fisher’s exact tests were conducted to assess significant peaks and troughs in prevalence during the 2008–2021 study period. Autocorrelation was observed when provirus data was plotted separately based on source (tissue or blood), so no pairwise tests were conducted for the time series. The spatial clustering of exposure to FeLV was assessed using the Kulldorff spatial scan test^66^. The level of agreement in FeLV provirus qPCR results between tissue types was assessed by calculating Cohen’s kappa coefficient in paired tissues. Statistical analyses were performed with SAS (version 9.4) and JMP Pro 16.0 (SAS Institute, Cary, NC, USA) and “rstatix” and “stats” R statistical software packages (v4.3.1)^67,68^. Results with *p* < 0.05 were considered statistically significant. Cluster detection was performed using the software SatScan^69^.

Both our research methodology and the lynx management were approved by the Andalusia Regional Government of Environment (11/2006, 01/2009, 07/2013, 03/2018 and 9/2021). All sanitary/health protocols were designed by the Advisory Group for health aspects of the Iberian Lynx Captive Breeding Program (involving National Authorities of Spanish and Portuguese Ministries of Environment, Spanish Regional Governments and external advisory experts)^33^. This study did not involve the intentional killing of any individual. Iberian lynxes were sampled by authorized veterinarians of the LIFE program, following routine procedures on live/dead lynxes before the design of this study, in compliance with Ethical Principles in Animal Research.

## Data Availability

Data from the study are available from the corresponding authors upon reasonable request.
